# Circular RNAs regulate cancer-related signaling pathways and serve as potential diagnostic biomarkers for human cancers

**DOI:** 10.1186/s12935-021-02017-4

**Published:** 2021-06-23

**Authors:** Pranavi Garlapati, Jinjie Ling, Paul J. Chiao, Jie Fu

**Affiliations:** 1 Department of Molecular and Cellular Oncology, The University of Texas MD Anderson Cancer Center, Houston, TX 77030 USA; 2 Columbia University Vagelos College of Physicians and Surgeons, New York, NY 10032 USA

**Keywords:** Circular RNA, Signaling pathways, Human cancer, Biomarkers, miRNA sponging

## Abstract

Circular RNAs (circRNAs) are RNAs that have an important role in various pathological processes, including cancer. After the usage of high-throughput RNA sequencing, many circRNAs were found to be differentially expressed in various cancer cell lines and regulate cell signaling pathways by modulating particular gene expressions. Understanding their role in these pathways and what cancers they are found in can set the stage for identifying diagnostic and prognostic biomarkers and therapeutic targets of cancer. This paper will discuss which circRNAs are found in different cancers and what mechanisms they use to upregulate or downregulate certain cellular components.

## Background

Circular RNAs (circRNAs) are a novel class of endogenous covalently closed RNA molecules created by back splicing of exons from a single pre-mRNA. They are formed by joining the 3′ end of a downstream exon to the 5′ end of an upstream exon [1]. CircRNAs are found to be predominantly located in the cytoplasm and highly resistant to degradation due to their structure, where the linear ends are not accessible. The biogenesis of circRNAs is known to be highly regulated by intronic complementary sequences and splicing factors [2].

Sanger et al. in 1976, was the first to elucidate that single-stranded viroids are covalently closed circular RNA molecules in plants [3]. Initially, this noncoding RNA was thought to be a result of a post-transcriptional error, but in recent years, the advent of novel sequencing technologies advanced our understanding that CircRNAs represent a new type of alternative splicing of a pre-mRNA. Emerging evidence shows that this non-coding RNA has an important role in biological processes and disease development [4]. Studies have shown that circRNAs are dysregulated in various diseases such as neurodegenerative disease, cardiovascular disease, viral infection, and most important to this paper, cancer [5-8]. Over 30,000 circRNAs have been identified as of 2018, and the discovery of new ones continues to progress. There are 4 main types of circRNAs: intergenic circRNAs, exon–intron circRNAs, circRNA (ecircRNA), and circular intronic RNA (ciRNA), all of which can play key roles in regulating cellular functions [9, 10]. As more circRNAs continue to be found, it becomes increasingly important to define their function in cancer. The characteristics of a circ-RNA such as its stability, distribution, and specific expression in various cell or tissue types give rise to its functional role. In cancer, circRNAs have been shown to modulate cancer growth, metastasis, Tumor Node Metastasis (TNM) stage, and drug resistance [11-13]. Several circRNAs have been reported to increase cell proliferation in cancer cells. Examples include circ0005276 in prostate cancer cells through the activation of X-linked inhibitor of apoptosis protein (XIAP) and circVAPA in HCC cells through the activation of prosaposin (PSAP) [14, 15]. Certain circRNAs can also inhibit cancer cell proliferation like Circular RNA YAP1 in gastric cancer cells [16]. CircRNA also regulates the invasion and metastasis of cancer cells: examples of this are CircRIMS in gastric cancer cells and hsa_circ_0023404 in non-small cell lung cancer (NSCLC) [17, 18]. Another function of circRNA is controlling the cell cycle. circ-MDM2 in CRC regulates MDM2 leading to p53 suppression and defects in G_1_-S progression [19]. Overall, circRNAs play a very important role in cancer.

### CircRNAs as biomarkers

circRNA provides an important biomarker for cancer due to a few unique reasons. First, circRNAs are highly resistant to degradation by exonucleases and extremely stable because they do not have 5′ or 3′ prime ends and therefore have a high degree of tissue and disease specificity [20]. Second, circRNAs are found in all cancer cell tissues, solid tumors, peripheral blood, exosomes, and other body fluids such as saliva, plasma, and serum [21]. A recent study with over 1195 plasma samples showed that hsa_circ_0007750, and hsa_circ_0139897 levels were significantly higher in patients with hepatocellular carcinoma (HCC) than healthy controls as well as patients with other diseases such as hepatitis B or HBV-related liver cirrhosis, indicating its specificity to cancer [22]. Because they are resistant to degradation and present in body fluids, circRNAs are the perfect candidate for noninvasive liquid biopsy and therefore have a high diagnostic potential. An example of this can be seen in a recent study where circ-KLDHC10 in serum samples was successfully used to distinguish patients with CRC from those without CRC [20]. In Lung adenocarcinoma, hsa_circ_0013958 was significantly upregulated and also correlated with lymphatic metastasis and tumor-node-metastasis (TNM) stage, implicating it as an important biomarker for cancer [23]. Most important to this paper is that circRNAs are the optimal biomarkers for cancer because they regulate cancer signaling pathways. Many circRNAs to date have been shown to play important roles in cancer signaling pathways by upregulating or downregulating important downstream proteins in pathways such as Wnt/β-Catenin, PI3K/Akt, MAPK/ERK, etc. [25, 26]. The role that circRNAs play in cancer, specifically in cancer signaling pathways, will be emphasized in this paper.

### Function of CircRNAs

Two key points make circ-RNAs a very important area of study, especially the role they play in cancer. First, it has been shown that circRNAs are expressed in every stage of cellular development, with an acute specificity [25]. Second, it has also been shown that the type of circRNA and its level of expression varies with the tumor type, tumor size, and metastatic ability [26]. Thus, circRNA has a great potential to serve as a biomarker in cancer. Also, there are four mechanisms by which circRNA can act in a cell. First, circRNAs can bind to RNA-binding proteins (RBPs), competitively inhibiting protein-active entities in a sequence-based way, possibly impacting cell proliferation and development [27, 28]. The second way in which circRNAs can act is by regulating gene transcription or even post-transcription in *cis*, and studies show that Exon–intron circular RNAs and circRNAs located in the nucleus usually regulate genes at the transcriptional level [29, 30]. The third mechanism by which circRNAs act, discovered very recently, is by translating proteins [31, 32]. The last and most important function of circRNAs is the ability of circRNAs to serve as MicroRNAs (miRNAs) sponges to regulate gene expression. miRNAs are a family of non-coding RNAs that regulate gene expression by pairing to specific sequences on target sites of mRNAs and usually causing mRNA degradation or repression of translation [33].

Many circRNAs have been shown to regulate miRNA complexes by acting as competitive endogenous RNA (ceRNA). ciRS-7, the first circRNA shown to function through sponging a miRNA, has been implicated in many cancers such as, colorectal cancer (CRC), non-small cell lung cancer (NSCLC), and esophageal squamous cell carcinoma (ESCC) [34, 35]. By binding to miRNA target sites as a miRNA sponge, circRNA can act as a competitive inhibitor and prevent miRNA from binding to its target genes and proteins. This can cause severe dysregulation of miRNA in the cell, especially if the miRNA had tumor-suppressive functions [36]. The role of circRNAs as a miRNA sponge in tumor progression and regression is a topic of great interest, and several papers have been published on that topic [37-39]. However, little is known about the mechanism by which circRNAs regulate cell signaling pathways in distinct human cancers. Recent and relevant publications focusing on how circRNAs modulate cell signaling pathways through the sponging of miRNA are discussed in this review. The various circRNAs in the mentioned pathways are shown in Table 1. Understanding which specific circRNAs are implicated in each cancer and the network through which they act can be important for the detection and treatment of cancer.

**Table 1:** CircRNAs and target miRNAs in Pathways: All circRNAs mentioned are grouped by the pathway they regulate (NF-κB, MAPK/ERK, JNK, PI3K, HIF, Wnt, VEGF) through upregulation or downregulation

Pathways	Upregulation	Downregulation
MAPK	[41] hsa_circ_0003204 [42] circ-MAPK4/miR-125a-3p [43] hsa_circ_0002124 [44] circ_LRIG3/miR-223-3p [45] circRNA CCDC66 [46] ciRS-7/miR-7 [47] circCEP12/miR‐145‐5p	
PI3K	[53] hsa_circ_0067934 [54] Hsa_circ_0002577/miR-625-5p	[55] circRHOBTB3 [56] hsa-circ-0072309/miR-100 [57] circCDK13 [58] hsa_circ_0004018
NF-κB	[60] circGLIS2/miR-671 [62] ciRS‐7/miR-7 [63] cZNF292 [64] circANKRD12	[61] circRNA-000911/miR-449a [65] circC3P1/miR-4641
JNK	[69] circ-102004 [69] CircMAN2B2/miR‐145 [70] CircUBAP2 [71] circLPAR3/miR-198	[68] circ-ZKSCAN/miR-330-5p
Wnt	[74] circβ-catenin [76] hsa_circ_000984 [77] circRNA_102171 [80] circ_001569	[73, 78] cir-ITCH/miR-17 [75] hsa_circ_009361/ miR-582 [78, 79] circ-ITCH/miR-214
HIF	[82] circRNF20/miR-487a [83] circSLC25A16/miR-488-3p [84] Circ-Erbin/miR-125a-5p [84] Circ-Erbin/miR-138-5p [85] circDENND2A/miR-625-5p [86] hsa-circ-0046600/miR-640 [87] circRNA_100859/miR-217 [89] circDENND4C [90] circ-0000977/miR-153 [91] circ-PIP5K1A/miR-600 [93] circ-HIPK3/miR-338-3p	[88] circFAM120A [92] circ-CDR1as/miR-135b-5p
VEGF	[97] circ_001621/miR-578 [98] circMYLK/miR-29a [99] circPVT1 [100] hsa_circ_0000096 [101] circPRRC2A/miR-514a-5p [101] circPRRC2A/ miR-6776-5p	

### CircRNAs are involved in the dysregulated signaling pathways in cancers

#### MAPK pathway

**Fig. 1:**
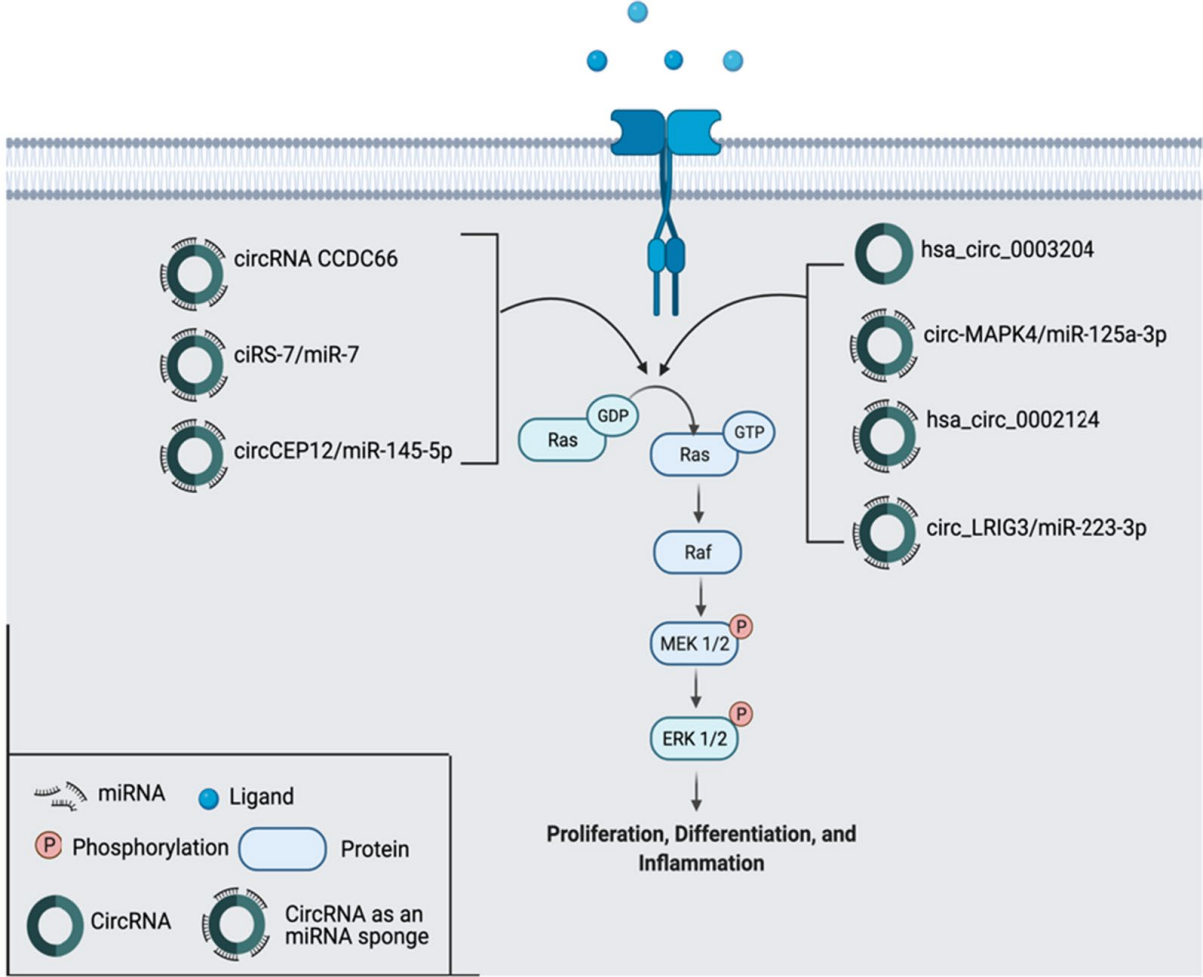
CircRNAs in MAPK pathway. MAPK Pathway plays an important role in tumorigenesis. There are 7 circRNAs (hsa_circ_0003204, circ-MAPK4/miR-125a-3p, hsa_circ_0002124, circ_LRIG3/miR-223-3p, circRNA CCDC66, ciRS-7/miR-7, circCEP12/miR‐145‐5p) that are significantly upregulated. 6 circRNAs regulate this pathway through miRNA sponging

The mitogen-activated protein kinase (MAPK) pathway plays a key role in cell differentiation and proliferation and is a commonly mutated cell signaling pathway in cancer. The MAPK pathway is activated first through the binding of a growth factor such as epidermal growth factor (EGF) to mutated Ras GTPase, which then recruits Raf kinases to the plasma, followed by the activation of downstream MAP kinase kinase (MEK 1/2) and eventually the extracellular signal-regulated kinase (ERK1/2) cascade (Fig. 1). The activation of this cascade leads to the transcription of various cytokines leading to the proinflammatory environment necessary for tumorigenesis [40].

Dysregulation of the MAPK pathway due to aberrant circRNA expression has been shown to activate this pathway in many cancer types. For example, in Cervical cancer (CC), hsa_circ_0003204 was found to be overexpressed, directly causing the activation of the MAPK pathway and significantly increasing tumor weight [41]. In another study considering the implications of circRNAs in gliomas, circ-MAPK4 was reported to promote glioma cell proliferation both in vivo and through upregulation of the MAPK axis. It was found that circ-MAPK4 positively regulated the MAPK pathway by acting as a sponge to miR-125a-3p, a miRNA that plays a tumor-suppressive function by inhibiting the MAPK pathway. Therefore, circ-MAPK4 can be seen as a therapeutic target as knockdown of circ-MAPK4 allows miR-125a-3p to regain its tumor suppressive function [42]. In Hepatocellular Carcinoma (HCC), it was found that hsa_circ_0002124 expression was much higher in Hepatocellular Carcinoma tissues than pericarcinomatous tissues, and the knockdown of this specific circRNA resulted in the decrease of cell proliferation and increase in apoptotic activity. Furthermore, hsa_circ_0002124 was hypothesized to act as a miRNA sponge, upregulating key proteins in the MAPK pathway such as ERK, c-Jun N-terminal kinase (JNK), and p38 [43]. Another study investigating circRNA function in HCC discussed the mechanism by which circ_LRIG3 increased cell proliferation and metastasis through targeting tumor suppressor miR-223-3p to activate the MAPK/ERK pathway. Knockdown of circ_LRIG3 inhibited the tumorigenic effects and induced apoptosis of HCC tissues, marking it as a valuable therapeutic target [44]. The overactivation of the MAPK pathway is also found in lung adenocarcinoma (LUAD). In this cancer type, circRNA CCDC66 induced EGFR overexpression, directly increasing the effects of the MAPK pathway. circRNA CCDC66 has a broadband effect because it can act as a sponge for multiple miRNAs resulting in the expression of many target genes [45]. Another very important circRNA implicated in multiple cancers is ciRS-7. ciRS-7 was found to act on the MAPK pathway by sponging miR-7 and suppressing its activity to increase the expression of miR-7 target genes [46]. miR-7 has shown anti-proliferative effects in many cancers through its target oncogenes such as AKT in hepatocellular carcinoma, EGFR in glioblastoma (GBM), and PAX6 in colorectal cancer [47-49]. ciRS-7 has over 70 binding sites for miR-7 and functions as a competitive inhibitor, therefore causing the expression of all of the above oncogenes. Thus, targeting ciRS-7 is very promising in terms of treatment as its effects are widespread in many cancer types due to it sponging miR-7. In bladder cancer, circCEP12 was overexpressed and found to promote the MAPK pathway and its related proteins including *MYD88*, p38, and ERK through the sponging of miR‐145‐5p [50]. The MAPK pathway is a key mutated pathway in many cancers as demonstrated above, and the circRNAs mentioned all play significant roles in this pathway. The circRNAs regulating the MAPK pathway are clearly shown in Fig. 1.

#### PI3K pathway

**Fig. 2:**
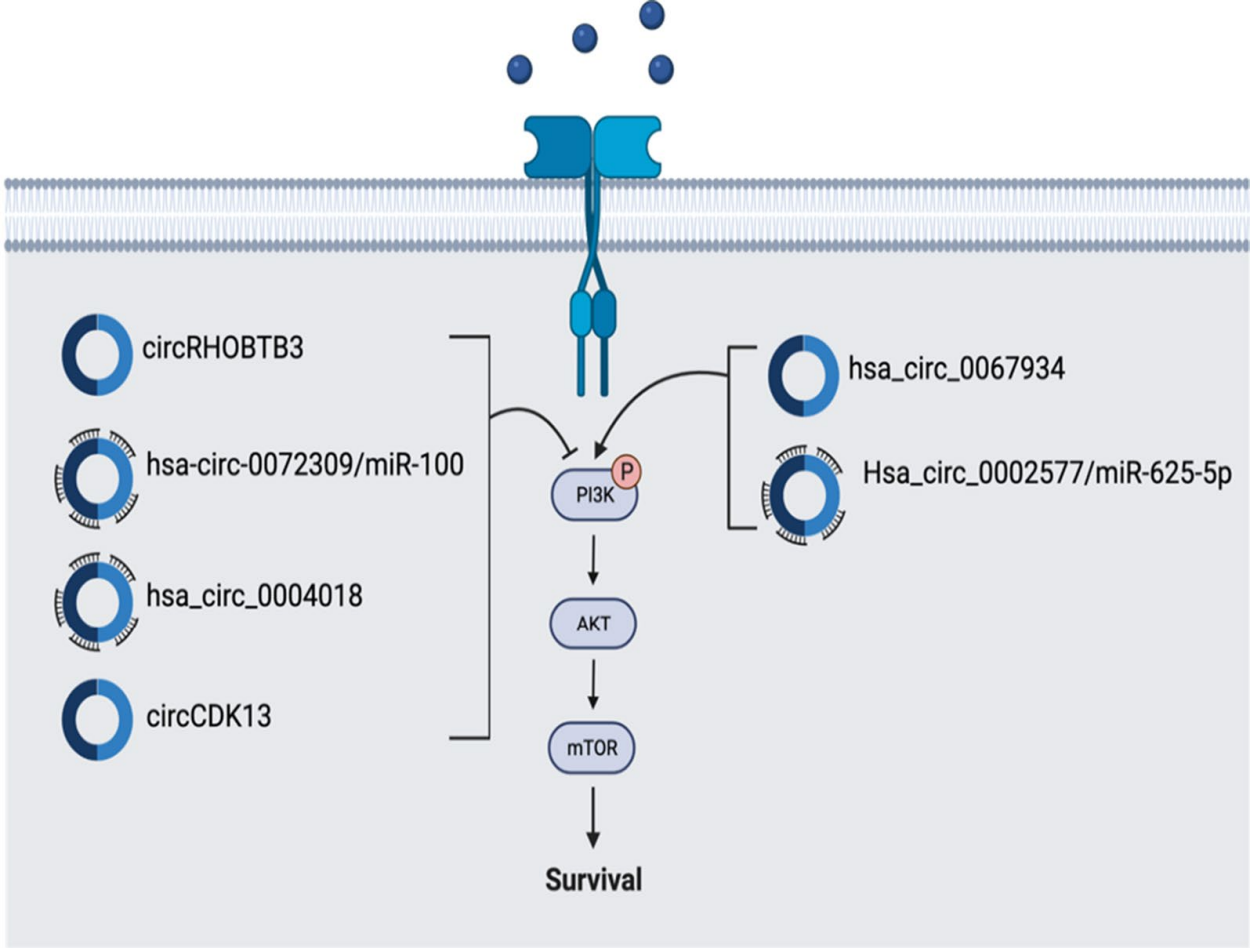
CircRNAs in PI3K pathway. Activation of PI3K Pathway is involved in cancer pathogenesis. There are 2 circRNAs (hsa_circ_0067934 Hsa_circ_0002577/miR-625-5p) that are overexpressed and 4 circRNAs (circRHOBTB3, hsa-circ-0072309/miR-100. circCDK13, hsa_circ_0004018) that show reduced expression. 5 circRNAs regulate this pathway through miRNA sponging

The Phosphoinositide 3-kinase (PI3K) pathway has been shown to play critical roles in cell metabolism, growth, and proliferation in cancer. Receptor Tyrosine Kinases (RTKs) in the cell membrane are activated by exogenous signals in the PI3K pathway. The activation of the RTKs causes bound PI3K to convert Phosphatidylinositol 4,5-bisphosphate (PIP2) into Phosphatidylinositol (3,4,5)-trisphosphate (PIP3) and subsequently causes the downstream activation of Protein kinase B (Akt) and mechanistic target of rapamycin (mTOR) (Fig. 2) [51]. The PI3K pathway is dysregulated in almost all human cancers including pancreatic cancer, colorectal cancer, breast cancer, etc. [52].

Recent studies have shown that circRNAs have caused the activation of the PI3K/AKT/mTOR pathway in a wide range of cancers (Fig. 2). Expression pattern in GBM cell and tissue lines showed that hsa_circ_0067934 was overexpressed and the knockdown of hsa_circ_0067934 significantly decreased tumor growth and cell proliferation. In addition, the study also showed that hsa_circ_0067934 promoted cancer cell proliferation by upregulating the PI3K-AKT pathway [53]. Another study concerning endometrial cancer showed the role that Hsa_circ_0002577 plays in cell proliferation, migration, and invasion. By sponging of miR-625-5p, Hsa_circ_0002577 induced the expression of IGF1R, the downstream target of miR-625-5p, and activated the PI3K/Akt pathway. Strikingly, the Knockdown of Hsa_circ_0002577 significantly decreased tumor growth and metastasis, marking it as a potential therapeutic target [54]. While the aforementioned circRNAs contribute to cell proliferation and metastasis, several circRNAs have been shown to harbor an opposing function by inhibiting cancer progression through the downregulation of the PI3K-AKT pathway. For example, a very recent study exemplified that in ovarian cancer, circRHOBTB3 inhibits tumor development through the suppression of the PI3K-AKT pathway [55]. Similarly, in renal carcinoma (RCC), hsa-circ-0072309 plays an anti-tumor role through the sponging of miR-100. This eventually causes the deactivation of PI3K/AKT/mTOR pathway with an increase in cell apoptosis. It was also mentioned that hsa-circ-0072309 expression was reduced in the cancerous cell lines, but its overproduction resulted in blocking of the PI3K/AKT/mTOR pathway and the inhibition of cell proliferation, migration, and invasion [56]. In hepatocellular carcinoma, circCDK13 prevented the migration and invasion of the liver cancer cells while also altering cell cycle progression through the inactivation of the PI3K/AKT/mTOR pathway. circCDK13 expression was also markedly decreased in the HCC cells [57]. Another circRNA that plays a similar role in hepatocellular carcinoma is hsa_circ_0004018, which is also severely under-expressed. hsa_circ_0004018 through the sponging of miRNA plays a role in regulating cell signaling pathways to inhibit cancer growth [58]. Strategies directed toward restoration of tumor-suppressive function of these circRNAs and manipulation of their regulatory activities on the specific signaling pathways may be clinically beneficial in limiting the development of cancers. Overall, The PI3K/AKT pathway has many circRNAs both inhibiting and activating it, so further research needs to show if these circRNAs have therapeutic potential for treatment. The circRNAs regulating the PI3K pathway are summarized in Fig. 2.

#### NF-κB pathway

**Fig. 3:**
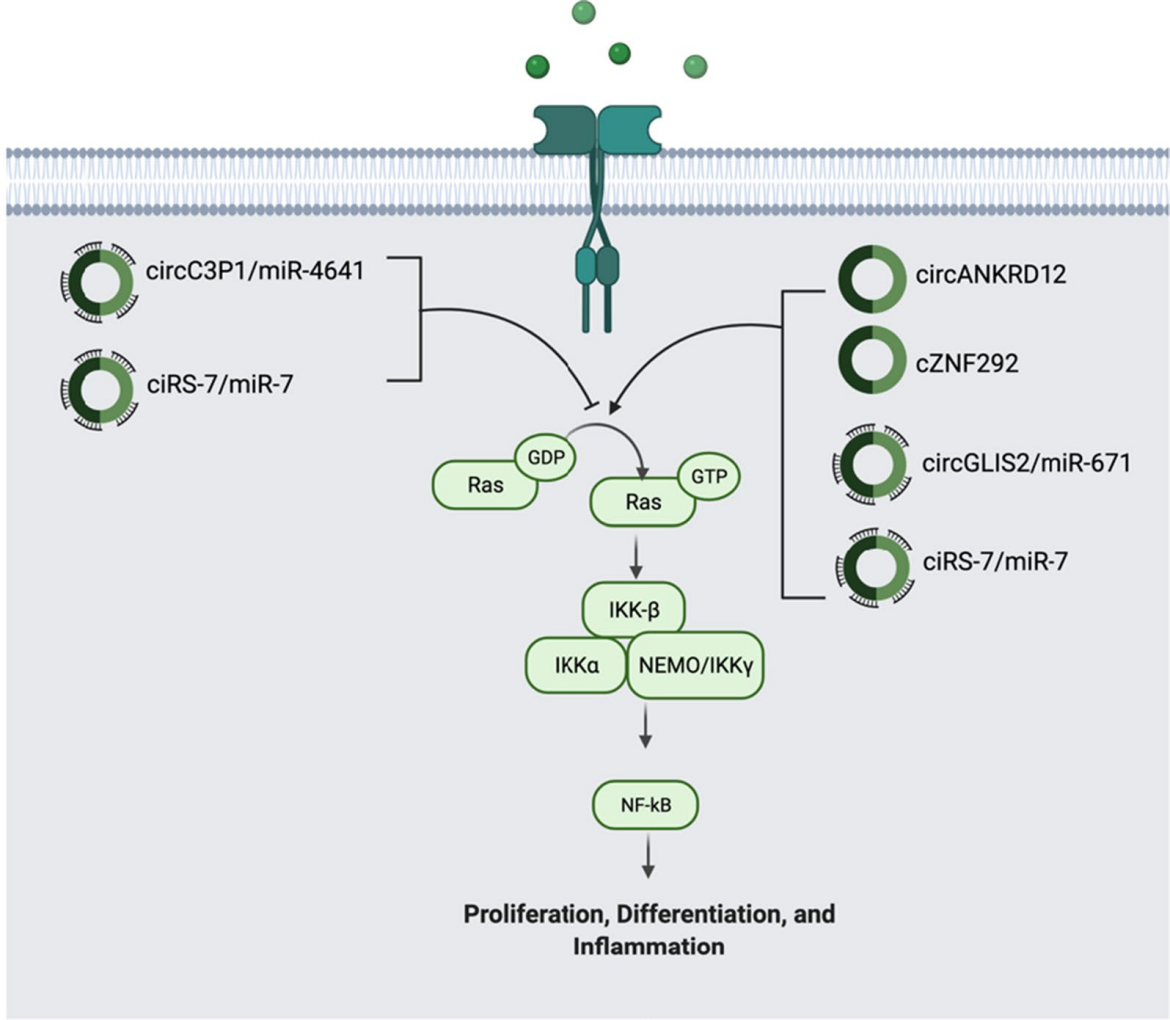
CircRNAs in NF-κB pathway. NF-κB controls inflammation, cancer cell proliferation and survival. There are 4 circRNAs (circGLIS2/miR-671, ciRS‐7/miR-7, cZNF292, and circANKRD12) that are significantly upregulated and 2 circRNAs (circRNA-000911/miR-449a and circC3P1/miR-4641) that are downregulated. Their functions coupled with miRNAs in the pathway are indicated in the figure

Nuclear Factor kappa-light-chain-enhancer (NF-κB) is an important transcription factor that plays diverse roles in cancer through controlling angiogenesis, proliferation and survival, EMT, cancer stem cell formation, and cell metabolism. NF-κB regulates the transcription of various cytokines, leading to cancer-related inflammation. Studies show that proinflammatory cytokines-mediated constitutive activation of NF-κB is required for mutant KRAS-driven tumorigenesis. The pathway of NF-κB is as follows: in response to a growth factor such as epidermal growth factor (EGF) or cytokines like tumor Necrosis Factor alpha (TNF-α) and Interleukin 1 alpha (IL-1α), the IκB kinase (IKK) complex is activated and NF-κB dimers can transport to the nucleus and induce the expression of target genes (Fig. 3) [59].

The role of circRNAs is to either upregulate or downregulate this pathway to leading to cancer progression or inhibition, respectively. circGLIS2 is shown to be involved in the regulation of cell motility in colorectal cancer cell lines. Via the sponging of miR-671, circGLIS2 was able to not only cause the cancer cells to acquire the ability to migrate, but also activate NF-κB signaling and induce chemokine-induced inflammation [60]. On the other hand, in breast cancer, circRNA-000911 plays an anti-oncogenic role. The miRNA associated with circRNA-000911 plays a tumorigenic role by promoting the NF-κB pathway while circRNA-000911 suppressed the NF-κB pathway. Decreased expression of circRNA-000911 and the increased expression of miR-449a leads to the activation of the NF-κB pathway, eventually leading to breast tumorigenesis and progression [61]. Another study investigated the role of circRNA in activating the NF-κB pathway in non-small cell lung cancer (NSCLC). ciRS‐7 was extremely upregulated while its target miRNA sponge miR-7, followed the opposite trend in cancer cell tissue compared to normal tissue. The highly expressed ciRS-7 was a prognostic factor that had correlations with larger tumor size and advanced histopathological grade. The highly expressed ciRS-7 also correlated with the high expression of RELA (also known as NF-κB-p65), indicating that ciRS-7 activated NF-κB to regulate the proliferation and invasion of NSCLC cells [62]. In glioma cell lines, cZNF292 upregulation led to the activation of many cancer-related cell signaling pathways including NF-κB. The silencing of cZNF292 also resulted in the downregulation of transcription factors like NF-κB, marking it as a potential therapeutic target [63]. Another study reported the effects of circANKRD12 in various cancers. circANKRD12 expression induced the alteration of the cell cycle as well as the appearance of an invasive phenotype in cancer cells. NF-κB was also markedly upregulated in addition to other cytokines and angiogenic factors, resulting in the inflammation and angiogenesis needed for cell invasion [64]. Lastly, in hepatocellular carcinoma, circRNA circC3P1 expression was negatively correlated with tumor size and progression. The method circC3P1 acts is through the sponging of miR-4641 and then PCK1 expression is needed to inhibit the NF-κB pathway. circC3P1 is essentially a tumor suppressor, and its overexpression could benefit treatment approaches [65]. The circRNAs regulating the NF-κB pathway are summarized in Fig. 3.

#### JNK pathway

**Fig. 4:**
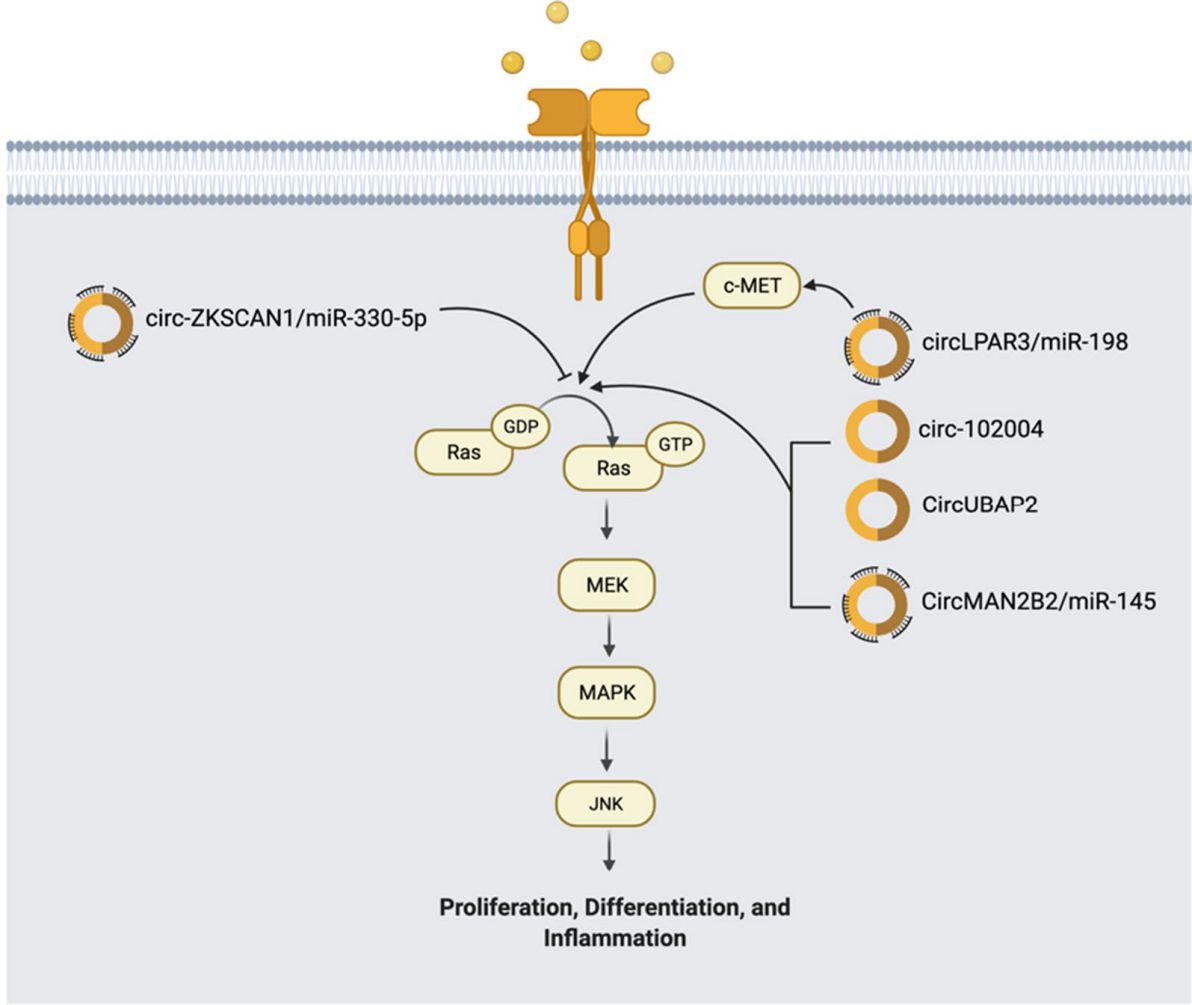
CircRNAs in JNK pathway. Dysregulation of JNK Pathway is involved in the cancer development. There are 4 circRNAs (circ-102004, CircMAN2B2/miR‐145, CircUBAP2, and circLPAR3/miR-198) that have significantly increased expression and 1 circRNA (circ-ZKSCAN/miR-330-5p) that has decreased expression. Some of them regulate this pathway through miRNA sponging

The c-Jun NH2-terminal kinase (JNK) Pathway has a dualistic role in cancer development because it has both pro and anti-tumor functions. Ras acts as a switch converting JNK from having an anti-tumor role to a pro-tumor role. JNK is usually activated by stress cytokines such as IL-1α and TNF-α [20]. Ras activates downstream effectors, MEK, MAPK kinase 4 or 7, and eventually JNK. JNK induces the transcription of Activator Protein-1 (AP-1), which is commonly implicated in cell proliferation and differentiation (Fig. 4). Dysregulation of the JNK pathway may increase cell proliferation [66].

circRNAs are shown to regulate the JNK pathway and play a key role in several cancers (Fig. 4). Circular RNA circ-102004, in prostate cancer, was shown to upregulate many pathways, including the JNK pathway. This increased cancer invasion and migration in the tissues and holds promise as a therapeutic target for treatment [67]. Another study detailing the effects of circular RNA in non-small cell lung cancer (NSCLC) explained that the activity of the JNK pathway severely decreases with the expression of circ-ZKSCAN. circ-ZKSCAN1 promoted the development of miR-330-5p and indirectly decreased JNK activity as well as MAPK activity [68]. A newly found circRNA, CircMAN2B2, acts as an oncogene in lung cancer and glioma. In gastric carcinoma (GC), CircMAN2B2 was found to promote gastric cancer cell growth and migration through the sponging of miR‐145. miR-145 plays a role in controlling both the PI3K/AKT and JNK pathways, so the sponging of miR-145 by CircMAN2B2 upregulates both pathways, leading to tumorigenesis [69]. CircUBAP2 is another circular RNA with a high expression that promotes the metastasis of lung adenocarcinoma through the upregulation of the JNK signaling pathway. CircUBAP2 silencing significantly inhibited JNK activity as well as ERK1/2 activity in the MAPK pathway [70]. In esophageal squamous cell carcinoma, circular RNA LPAR3 (circLPAR3) was found to be severely overexpressed both in vitro and in vivo promoting ESCC cell migration, invasion, and metastasis. circLPAR3 acts as a miRNA sponge to inhibit miR‐198. miR-198 is an important negative regulator of c‐MET kinase, a transmembrane receptor with phosphorylation activity controlled through the oncogene *MET*. C-MET serves as an on switch for pathways like RAS/MAPK, PI3K/Akt, and most importantly, STAT3/JNK, leading to cell proliferation and invasion. The sponging of miR‐198 by CircLPAR3 regulates MET expression leading to the tumorigenic effects described above [71]. Many circRNAs described in the JNK pathway seem to activate or inhibit its pro-tumorigenic role in cancer. It is unknown yet whether there are circRNAs that can activate the antitumorigenic role of the JNK pathway. The circRNAs regulating the JNK pathway are clearly shown in Fig. 4.

#### WNT pathway

**Fig. 5:**
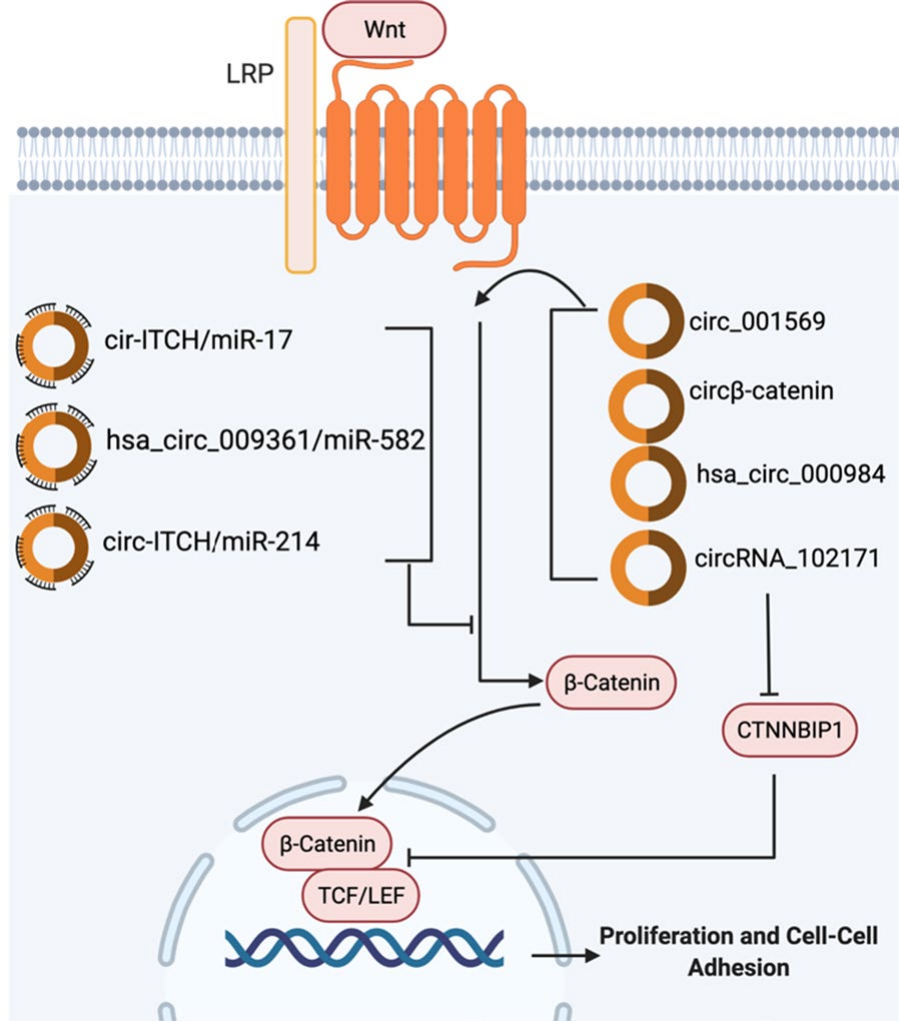
CircRNAs in Wnt pathway. Wnt/β-Catenin Pathway plays a critical role in cancer progression. There are 4 circRNAs (circβ-catenin, hsa_circ_000984, circRNA_102171, and circ_001569) that are significantly upregulated and 3 circRNAs (cir-ITCH/miR-17, hsa_circ_009361/ miR-582, and circ-ITCH/miR-214) that are downregulated. Their functions in regulating Wnt pathway are indicated in the figure

Wingless-related integration site (Wnt) signaling, a key signaling pathway regulating development, tissue homeostasis, and stemness, is tightly associated with cancer and has been most prominently described in colorectal cancer. The pathway starts with secreted Wnt ligands including Wnt3a and Wnt1 binding to frizzled (FZD) receptors and Low-density lipoprotein receptor-related protein (LRP) co-receptors and preventing β-catenin degradation. Stabilized β-catenin binds to LEF (lymphoid enhancer factor) and TCF (T-cell factor) regions to form an active transcriptional complex for transcription of several vital gene targets. The transcription of the target genes results in cell proliferation capabilities (Fig. 5). The Wnt pathway is commonly implicated in cancer as a critical regulator of tumorigenesis [72].

Many circRNAs have been shown to regulate Wnt signaling (Fig. 5). For example, cir-ITCH can act as a negative regulator of Wnt/β-catenin signaling. Interestingly, it is found that miR-17 can reverse this effect. In gastric cancer, cir-ITCH was shown to prevent tumorigenesis by sponging miR-17 to inhibit the Wnt/β-catenin signaling pathway [73]. circβ-catenin is found to be highly expressed in liver cancer tissues compared to normal tissues. Knockdown of circβ-catenin significantly suppressed tumor malignancy both in vitro and in vivo [74]. In colorectal cancer (CRC), through the sponging of tumor activator miR-582, hsa_circ_009361 upregulated APC2, an inhibitor in the activation of the Wnt and β-catenin pathway, where it forms the complex to destroy β-catenin. [75]. In non-small cell lung cancer (NSCLC), circRNA hsa_circ_000984 promotes the activation of the Wnt/β-catenin signaling pathway and therefore the proliferation, invasion, and EMT [76]. In thyroid cancer, CTNNBIP1 functions as a negative regulator of the Wnt/β-catenin pathway by decreasing the interaction between β-catenin and TCF/LEF to inhibit the expression of downstream target proteins. circRNA_102171 reduces the level of CTNNBIP1 in the nucleus, and therefore increasing the interaction between β-catenin and TCF/LEF, leading to the upregulation of the Wnt/β-catenin signaling pathway in thyroid cancer and promoting thyroid cancer cell proliferation, invasion, and migration [77]. Downregulation of circ-ITCH was observed in breast cancer (BC), and a new study showed that circ-ITCH could downregulate the Wnt/β-catenin signaling pathway by sponging miR-214 and miR-17 [78]. Similarly, in glioma cells, cir-ITCH plays a tumor-suppressive role by sponging miR-214 to increase ITCH protein expression and modulate the Wnt/b-catenin cascade. miR-214 is shown to activate the Wnt/β-catenin pathway through ITCH protein expression, so cir-ITCH inhibits that effect. Overexpression of cir-ITCH suppressed Wnt/β-catenin pathway, leading to the inhibition of glioma cell proliferation, migration, and invasion. Increasing the expression of cir-ITCH can be potentially used to block glioma tumor development [79]. circ_001569 was overexpressed in Osteosarcoma (OS) and was found to correlate with the OS tumor progression. Silencing of circ_001569 led to a decrease of β-catenin expression which decreased the overall activity of the Wnt/β-catenin signaling pathway. This suggests that circ_001569 activates the progression of OS through the activation of the Wnt/β-catenin signaling pathway [80]. Much still needs to be known about the role circRNAs play in Wnt signaling, but currently, the mentioned circRNAs hold promise as biomarkers in several cancers. The circRNAs regulating the Wnt pathway are clearly shown in Fig. 5.

#### HIF pathway

**Fig. 6:**
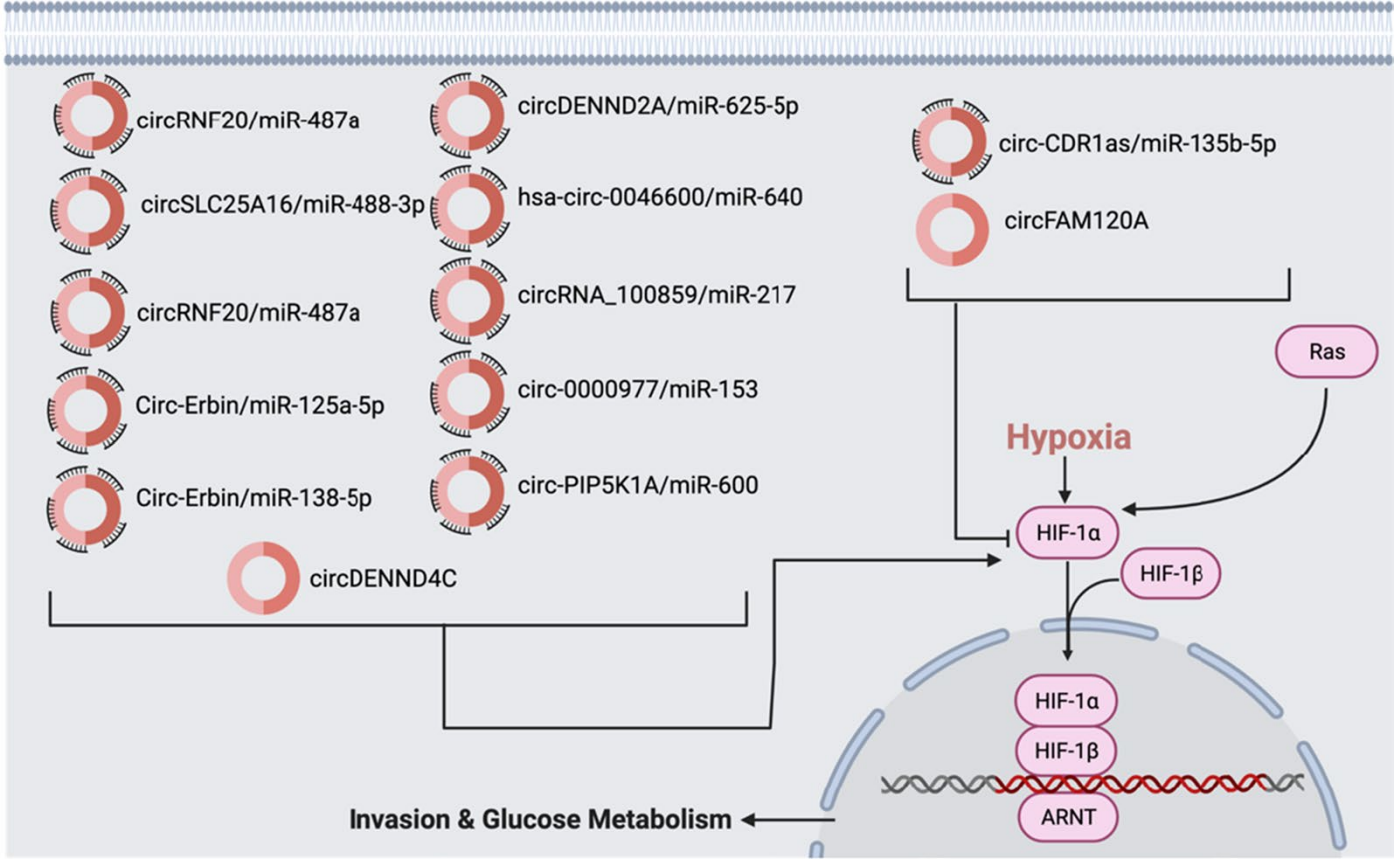
CircRNAs in HIF pathway. HIF-1 activation is associated with increased tumor growth. There are 11 circRNAs (circRNF20/miR-487a, circSLC25A16/miR-488-3p, Circ-Erbin/miR-125a-5p, Circ-Erbin/miR-138-5p, circDENND2A/miR-625-5p, hsa-circ-0046600/miR-640, circRNA_100859/miR-217, circDENND4C, circ-0000977/miR-153, circ-PIP5K1A/miR-600, circ-HIPK3/miR-338-3p) that are significantly upregulated and 2 circRNAs (circFAM120A and circ-CDR1as/miR-135b-5p) that are downregulated. Most of these circRNAs regulate this pathway through miRNA sponging

Activated Hypoxia-Inducible Factor (HIF)-1 plays an important role in the response of the tumor cells. Because of changes in oxygen, HIF-1a activates the transcription of over 100 downstream genes involved in controlling the metabolism of glucose, proliferation, cell migration, and angiogenesis to regulate vital biological processes required for tumor survival. HIF-1, as a part of the HIF pathway, can promote tumor metastasis into distant and oxygenated tissues through the activation of oncogenic growth factors such as EGF and transforming growth factor beta3 (TGF-*β*3) (Fig. 6). The activation of PI3K or the MAPK pathway can upregulate the HIF-1*α* protein expression or hypoxia, the deprivation of oxygen in cells, can also activate HIF-1α [81].

circRNF20 (hsa_circ_0087784) was found to be upregulated in Breast Cancer (BC) cell samples in a recent study. circRNF20 acts as a miRNA sponge to miR-487a. miR-487a targeted the 3’-UTR of HIF-1α to induce the Warburg effect, which is a distinctive cellular metabolic mechanism in cancer cells that causes long-term tumor cell survival [82]. In NSCLC cells, circSLC25A16 was upregulated and was also associated with a dismal prognosis. The study revealed that circSLC25A16 increased the glycolysis, survival, and proliferation of NSCLC cells. It was also revealed that circSLC25A16 could target miRNAs as a miRNA sponge, especially miR-488-3p. The study showed the potential of HIF-1α serving as the target of the circSLC25A16/miR-488-3p axis. HIF-1α then facilitated the expression of LDHA, which in turn promoted aerobic glycolysis, causing the growth and proliferation of NSCLC cells [83]. In colorectal cancer, Circ-Erbin was shown to be highly expressed and was associated with proliferation, invasion, and metastasis of CRC cells both in vitro and in vivo through increasing angiogenesis and inducing HIF-1α expression. Mechanistically, circ-ERBIN was shown to promote angiogenesis and metastasis of CRC by acting as a miRNA sponge to both miR-125a-5p and miR-138-5p, which act together to increase the expression of the eIF4E-binding protein 1 (4EBP-1). 4EBP-1 enhances the expression of HIF-1α and subsequently, the activation of the HIF-1α pathway [84]. Furthermore, hypoxia activated the expression of circDENND2A and consequently promoted the invasion of glioma cells. The mechanism through which circDENND2A functions is by sponging miR-625-5p. circDENND2A was also needed for the hypoxia-induced malignancy of glioma cells, marking it as an important therapeutic target [85]. In hepatocellular carcinoma, hsa-circ-0046600 was found to be significantly overexpressed in tumor tissues than that in adjacent normal tissues. hsa-circ-0046600 was associated with tumor size, stage, invasion, and angiogenesis. hsa-circ-0046600 promotes the expression of HIF-1α by targeting miR-640 as a miRNA sponge and consequently affecting the tumorigenesis of liver cancer cells [86]. In a recent study, circRNA_100859 was overexpressed in colon cancer cells and promoted cell proliferation and invasion, and simultaneously inhibited cell apoptosis. circRNA_100859 functions as a miRNA sponge by competitively binding to miR-217. miR-217 directly targets HIF-1α, so circRNA_100859 was able to inhibit the tumor-suppressive effects of miR-217. circRNA_100859-miR-217-HIF-1α axis was associated with Tumor-Node-Metastasis stage and KRAS mutations, both of which are important to the potential of a tumor to metastasize. circRNA_100859 also showed high prognostic and diagnostic value for patients with colon cancer and can act as a potential biomarker and therapeutic target. circRNA_100859 functions as an oncogene in colon cancer by sponging the miR-217-HIF-1α pathway [87]. Similarly, in lung adenocarcinoma tissues, HIF-1α expressions were significantly increased as compared to the adjacent normal lung tissues. This provides a premise in suggesting the presence of tumor hypoxia during the progression of lung adenocarcinoma. circFAM120A was downregulated in the hypoxic cancer cells, showing it may have tumor-suppressing activity [88]. In breast cancer (BC) cells, circDENND4C was increased and upregulated under hypoxic conditions. Silencing HIF1α reduced the expression of circDENND4C. In addition, knocking-down circDENND4C inhibited the survival and invasion of breast cancer cells in hypoxic conditions [89]. Another recent study showed that the circ-0000977 was induced by hypoxia in pancreatic cancer cells through the sponging of miR-153. This sponging effect causes the HIF1α-induced immune escape of pancreatic cancer cells through the mechanism of targeting HIF1α/ADAM10. HIF1 and ADAM10 are downstream target proteins of miR-153 and the sponging of miR-153 counteracts the tumor-suppressive role miR-153 plays [90]. In NSCLC cells, circ-PIP5K1A was overexpressed. The circ-PIP5K1A/miR-600/HIF1α axis caused proliferation and invasion of NSCLC cells in the study. Specifically, circPIP5K1A is shown to act as a miR-600 sponge to facilitate proliferation and metastasis of NSCLC cells by activating HIF-1α. This offers circ-PIP5K1A as a potential biomarker and target for treatment in NSCLC [91]. In patients with ovarian cancer, CDR1as expression was significantly lower than in patients without ovarian cancer. CDR1as overexpression, therefore, is shown to inhibit the proliferation, migration, and invasion, of ovarian cancer cells. circ-CDR1as acted as a sponge of miR-135b-5p to increase the expression of hypoxia-inducible factor 1-alpha inhibitor (HIF1AN), the inhibitor of HIF-1α. circ-CDR1as, therefore, exerts an inhibitory role on proliferation and has a tumor-suppressive function in ovarian cancer cells [92]. Another study revealed that circ-HIPK3 expression was significantly increased in cervical cancer (CC) cells. Silencing of circ-HIPK3 repressed progression and metastasis of CC cells, while also inducing apoptosis. The mechanism through which circ-HIPK3 functions is through sponging miR-338-3p [93]. More research needs to be done in regards to how to inhibit these circRNAs from activating the HIF pathway. The circRNAs regulating the HIF pathway are summarized in Fig. 6.

#### VEGF pathway

**Fig. 7:**
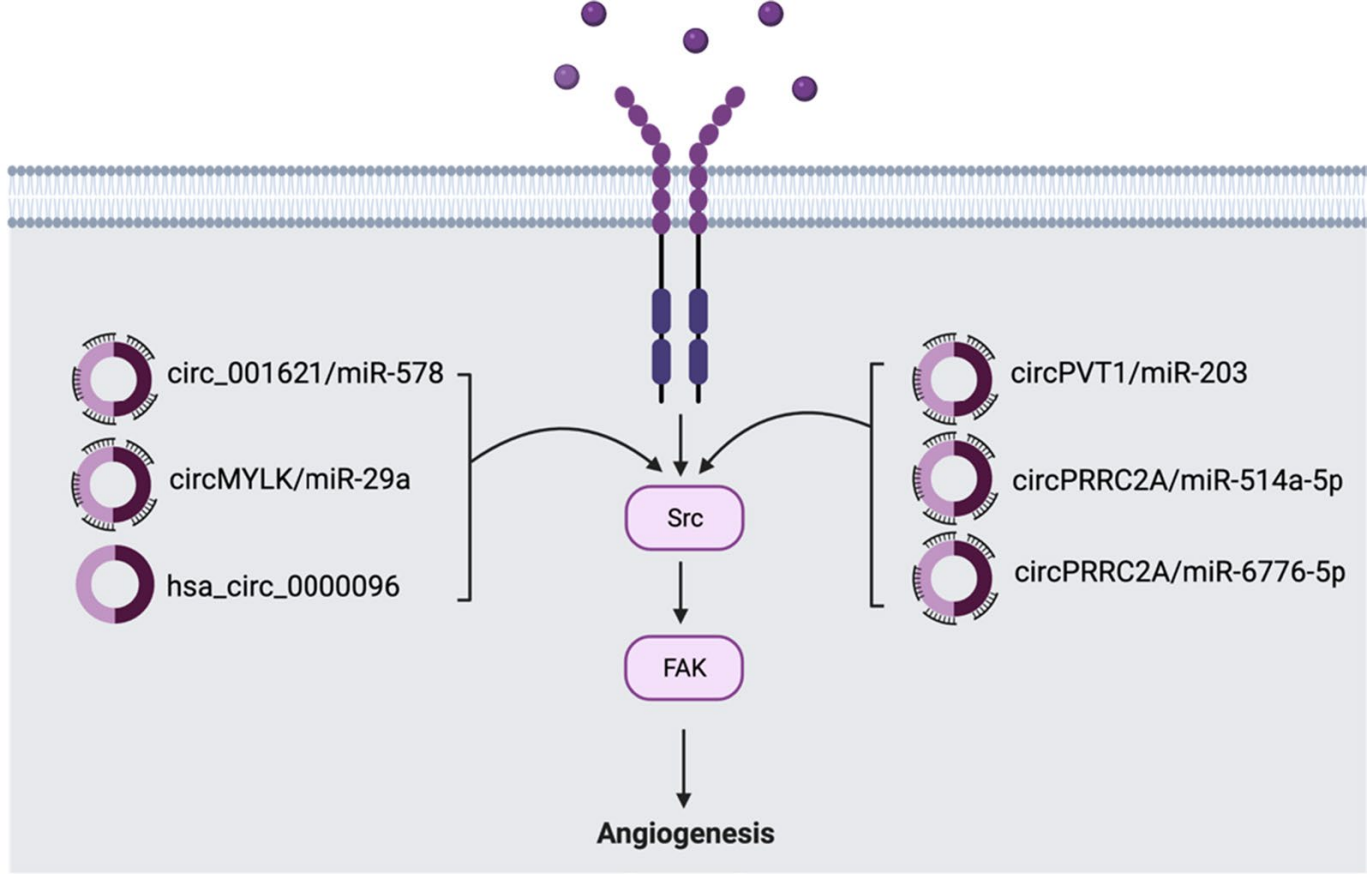
CircRNAs in VEGF pathway. VEGF Pathway plays an important role in angiogenesis and tumorigenesis. There are 6 circRNAs (circ_001621/miR-578, circMYLK/miR-29a, circPVT1, hsa_circ_0000096, circPRRC2A/miR-514a-5p, and circPRRC2A/ miR-6776-5p) that are significantly overexpressed. They regulate angiogenesis via sponging miRNA

Vascular endothelial growth factor (VEGF) is a member of a family of 6 related proteins that regulate the growth and differentiation of distinct parts of the vascular system. In cancer, VEGF can cause endothelial cell growth, survival, most importantly angiogenesis. Angiogenesis is a very important process in the development of cancer from localized tumors to large migrating tumors, and the VEGF pathway is particularly important in facilitating this process [94]. Activation of VEGF can induce the expression of downstream PI3K pathway proteins or MAPK proteins as well as a different set of proteins known as Src kinase (Src) and FAK Focal adhesion kinase (FAK). VEGF is first activated as an intracellular receptor by a ligand such as IL-1α and then angiogenesis is the response to subsequent activation of Src/FAK complex (Fig. 7) [95, 96].

Circular RNA circ_001621was recently implicated in osteosarcoma cells. circ_001621 was found to be significantly upregulated in osteosarcoma cells and was designated as an indicator of poor prognosis in patients with osteosarcoma. circ_001621 was shown to activate the VEGF pathway through the sponging of miR-578 in vitro and in vivo [97]. In bladder cancer, circMYLK was shown to promote angiogenesis through the upregulation of VEGF. The oncogenic role circMYLK plays is through the sponging of miR-29a. The direct target gene of miR-29a is VEGFA, so the sponging of miR-29a by circMYLK inhibits the tumor-suppressive role that it plays [98]. In hepatocellular carcinoma (HCC), circPVT1 overexpression was found and consequently resulted in a poor prognosis for patients with HCC. In vitro, silencing of circPVT1 expression significantly decreased tumor invasive properties. Initiation and progression of HCC was attained by circPVT1 functioning as a miRNA sponge to miR-203 and activating the VEGF pathway [99]. In gastric cancer, hsa_circ_0000096 was detected to be significantly upregulated. Silencing of hsa_circ_0000096 suppressed the invasion of gastric cancer cells and also decreased the expression of VEGF [100]. Another study discussing the role of circPRRC2A in renal adenocarcinoma suggested that circPRRC2A could play a role in activating the VEGF pathway. circPRRC2A was significantly upregulated in renal adenocarcinoma tissue and resulted in the invasion and metastasis of the cells. circPRRC2A also caused an elevation in the expression of VEGFA, and the mechanism by which it functioned was through the sponging of miR-514a-5p and miR-6776-5p, both of which played tumor-suppressive roles. Knockdown of circPRRC2A decreased tumor invasion and migration substantially, marking it as a potential biomarker and therapeutic target [101]. There is less information on the circRNAs that play a role in the VEGF pathway and looking ahead, it is worthwhile to research methods of preventing angiogenesis through the inhibition of the circRNAs mentioned above. The circRNAs regulating the VEGF pathway are clearly shown in Fig. 7.

## Discussion

By understanding canonical mutated cancer cell signaling pathways (NF-κB, MAPK/ERK, JNK, PI3K, HIF, Wnt, VEGF) and the circRNAs that have been implicated in these pathways, new treatment methods and rationale can be developed for cancer therapy. However, the mechanisms by which circRNAs function in these signaling pathways continues to be an active area of investigation. In this paper, most circRNAs exhibited a sponging method to inhibit or activate key cell signaling pathways, but there is not much information on other mechanisms through which these circRNAs act, such as modifying RNA or through epigenetic changes. Future studies should explore other mechanisms by which CircRNA acts other than sponging in greater depth. In addition, it is not known what causes the deregulation of circRNAs in cancer, and that’s another important factor to consider in the future. What is known is that circRNAs harbor promising potential as biomarkers for the signaling pathways mentioned and as novel targets for therapeutic treatment as they have important roles in cancer, such as cancer cell proliferation, invasion, and metastasis.

As mentioned, circRNAs show a lot of promise as diagnostic biomarkers as they are prevalent in common body fluids such as plasma, urine, blood, and saliva. Current techniques of cancer detection can be extremely invasive and highly expensive, but the development of techniques detecting circRNA clinically could combat this problem, especially through noninvasive liquid biopsy. Early detection of cancer is extremely important to prognosis, so exploring the diagnostic potential of circRNAs is of great importance. As a prognostic biomarker, circRNA has been known to play a role in cancer pathogenesis throughout this paper by miRNA sponging in cancer signaling pathways. Current chemotherapeutic drugs are not completely effective and tumor occurrence is extremely common. As a result, a prognostic biomarker that would predict tumor reoccurrence is of great significance. Many recent studies have demonstrated the use of circRNAs in the prediction of tumor reoccurrence. For example, high levels of circPVT1 in patients with GC had a significantly higher progression-free survival (PFC) than those that did not [25]. In addition, it was recently found that circRNAs were differentially found in radioresistant cancer cells. Radioresistance during the treatment of cancer is one of the biggest causes of tumor reoccurrence. In radioresistant esophageal cancer cells, 57 circRNAs were significantly upregulated, marking circRNAs as an important indicator of tumor resistance and occurrence. Noting the specific circRNAs that play a role in each cancer would be advantageous to the diagnosis and prognosis prediction of cancer [102].

For clinical treatment, the targeting of circRNAs could serve as second-line therapies for chemotherapy-resistant tumors. Silencing pro-tumor circRNAs can also be paired alongside targeted therapies and chemotherapies. For these synthetic lethality therapeutic approaches, further investigation into the toxicities of these combination therapies should be performed. Several studies have shown that circRNAs can play a role in the chemotherapeutic resistance of cancer. For example, over 68 circRNAs were found to be upregulated and 58 downregulated in PDAC cell lines contributing to Gemcitabine resistance. Furthermore, the silencing of 2 of the circRNAs that were more significantly expressed than others in PDAC cells restored gemcitabine sensitivity in treatment [103]. In Osteosarcoma, hsa_circ_001569 overexpression contributed to cisplatin resistance through the Wnt/β-catenin pathway, and similarly, in CRC, hsa_circ_0007031 and hsa_circ_0000504 overexpression promoted 5-FU resistance through the circRNA/miR-885-3p/AKT3 axis [104]. These drugs are the most commonly used drugs for cancer treatment for their respective cancers, so restoring sensitivity in resistant cancer cells is of great importance. As of now, there are no reports of circRNAs themselves being used as therapeutic targets. However, this warrants further studies that investigate other treatment methods such as surgical resection and radiotherapy along with the inhibition of circRNA. Another important factor to consider is the high rate of mutation of tumor-promoting proteins such as KRAS and EGFR in signaling pathways causing acquired resistance to targeted therapy. In cases like this, it may be useful to study methods of combination therapy by targeting both signaling pathways and circRNAs. In addition, studies that explore circRNAs potential in restoring sensitivity to treatment could be highly beneficial as it has been shown that long noncoding RNAs (lncRNAs) have been shown to do so in a recent study [105]. In future studies, it may be important to consider other manipulations of circRNAs such as the delivery of tumor suppressor circRNA through gene therapy or using the circRNAs as templates for tumor suppressive proteins as it was recently discovered that they can act as templates for protein expression [106]. It may also beneficial to consider the role of circRNAs as treatment vectors as they are extremely stable and have miRNA sponging functions. Delivering tumor suppressor proteins through circRNAs holds a lot of potentials as circRNAs have multiple binding sites for oncogenic proteins and as shown by this paper, play multiple roles in cancer signaling pathways. circRNAs play significant roles in cell signaling and disease, and our continued understanding of their biological functions may help us better exploit vulnerabilities in cancer and pathological processes.

In addition, cell signaling crosstalk and synergy have important implications for cancer treatment efficacy. Many pathways such as MAPK and PI3K pathways have similar upstream effectors and may work together to cause cell proliferation, migration, and invasion. Hence, elucidating circRNA networks and their multi-pathway consequences would enhance our current understanding of therapeutic targeting in cancer.

## Conclusion

CircRNAs have been implicated in many cancers and understanding their role and aberrant cell signaling pathways as possible biomarkers and therapeutic targets was the aim of this paper.

## Data Availability

Not applicable.

## Abbreviations

circRNAs: Circular RNAs
FAK: Focal adhesion kinase
ecircRNA: Exonic Circular RNA
ciRNA: Circular intronic RNA
EMT: Epithelial–mesenchymal transition
RBPs: RNA-binding proteins
miRNAs: MicroRNAs
ceRNA: Competitive endogenous RNA
CRC: Colorectal cancer
NSCLC: Non-small cell lung cancer
ESCC: Esophageal squamous cell carcinoma
MAPK: Mitogen-activated protein kinase
EGF: Epidermal growth factor
MEK ½: MAP kinase kinase
ERK1/2: Extracellular signal-regulated kinase
CC: Cervical cancer
HCC: Hepatocellular carcinoma
JNK: C-Jun N-terminal kinase
LUAD: Lung adenocarcinoma
PI3K: Phosphoinositide 3-kinase
RTKs: Receptor tyrosine kinases
PIP2: Phosphatidylinositol 4,5-bisphosphate
PIP3: Phosphatidylinositol (3,4,5)-trisphosphate
Akt: Protein kinase B
mTOR: Mechanistic target of rapamycin
GBM: Glioblastoma
NF-κB: Nuclear factor kappa-light-chain-enhancer
TNF-α: Tumour necrosis factor alpha
IL-1α: Interleukin 1 alpha
IKK: IκB kinase
RELA: p65
Ap-1: Activator protein-1
GC: Gastric carcinoma
Wnt: Wingless-related integration site
FZD: Frizzled
LRP: Low density lipoprotein receptor-related protein
LEF: Lymphoid enhancer factor
TCF: T-cell factor
OS: Osteosarcoma
HIF-1: Hypoxia-inducible factor (HIF)-1
TGF-*β*3: Transforming growth factor beta3
4EBP-1: The eIF4E-binding protein 1
TNM: Tumor-node-metastasis
HIF1AN: Hypoxia-inducible factor 1-alpha inhibitor
VEGF: Vascular endothelial growth factor
Src: Src kinase
FAK: Focal adhesion kinase
XIAP: X-linked inhibitor of apoptosis protein
PFC: Progression-free survival
PSAP: Prosaposin
lncRNAs: Long noncoding RNAs
